# Pox proteomics: mass spectrometry analysis and identification of Vaccinia virion proteins

**DOI:** 10.1186/1743-422X-3-10

**Published:** 2006-03-01

**Authors:** Jennifer D Yoder, Tsefang S Chen, Cliff R Gagnier, Srilakshmi Vemulapalli, Claudia S Maier, Dennis E Hruby

**Affiliations:** 1 Oregon State University, Department of Microbiology, 220 Nash Hall, Corvallis, OR 97331-3804, USA; 2 Oregon State University, Applied Biotechnology Program, 2082 Cordley Hall, Corvallis, OR 97331-8530, USA; 3 Oregon State University, Department of Chemistry, 153 Gilbert Hall, Corvallis, OR 97331-4003, USA

## Abstract

**Background:**

Although many vaccinia virus proteins have been identified and studied in detail, only a few studies have attempted a comprehensive survey of the protein composition of the vaccinia virion. These projects have identified the major proteins of the vaccinia virion, but little has been accomplished to identify the unknown or less abundant proteins. Obtaining a detailed knowledge of the viral proteome of vaccinia virus will be important for advancing our understanding of orthopoxvirus biology, and should facilitate the development of effective antiviral drugs and formulation of vaccines.

**Results:**

In order to accomplish this task, purified vaccinia virions were fractionated into a soluble protein enriched fraction (membrane proteins and lateral bodies) and an insoluble protein enriched fraction (virion cores). Each of these fractions was subjected to further fractionation by either sodium dodecyl sulfate-polyacrylamide gel electophoresis, or by reverse phase high performance liquid chromatography. The soluble and insoluble fractions were also analyzed directly with no further separation. The samples were prepared for mass spectrometry analysis by digestion with trypsin. Tryptic digests were analyzed by using either a matrix assisted laser desorption ionization time of flight tandem mass spectrometer, a quadrupole ion trap mass spectrometer, or a quadrupole-time of flight mass spectrometer (the latter two instruments were equipped with electrospray ionization sources). Proteins were identified by searching uninterpreted tandem mass spectra against a vaccinia virus protein database created by our lab and a non-redundant protein database.

**Conclusion:**

Sixty three vaccinia proteins were identified in the virion particle. The total number of peptides found for each protein ranged from 1 to 62, and the sequence coverage of the proteins ranged from 8.2% to 94.9%. Interestingly, two vaccinia open reading frames were confirmed as being expressed as novel proteins: E6R and L3L.

## Background

Variola virus (smallpox agent) and/or genetically-engineered orthopoxviruses are considered one of the most significant Category A pathogenic threats for malevolent use as potential agents of bioterrorism [1]. Due to the bioterrorism threat, there is a renewed public interest in the development of effective anti-poxvirus drug(s) and/or vaccines for use in treating or preventing human diseases caused by pathogenic poxviruses. Because the nucleotide sequence of the variola virus is approximately 90% identical with that of the vaccinia virus, VV [2], we hypothesize that VV can act as a model for variola. At present, there are no effective anti-orthopoxvirus drugs available, and the Dryvax vaccine used during the eradication campaign is not considered safe for general use, considering immuno-compromised people, and the complications associated with this live-attenuated vaccine.

Poxviruses, such as VV, are amongst the largest and most complex of the eukaryotic DNA viruses and are distinguished by replicating exclusively within the cytoplasmic compartment of infected cells [3]. VV regulates the expression of more than 250 viral gene products in a temporal fashion during the viral replicative cycle which results in at least four infectious forms all of which share the same intracellular mature virus (IMV) at their center which contains one membrane and a concave brick core. VV proteins are denoted by their corresponding open reading frame (ORF). The conventional designation of VV ORF consists of a Hind III DNA fragment (A-O), followed by the number of the ORF in that fragment (numbered left to right), and finally by the direction of the ORF (L or R).

Although the complete genome sequence of VV (strain Copenhagen) has been available for years [4], there has been little comprehensive proteomic analysis of the VV virion described so far. Jensen, *et al*. identified 13 major membrane and core proteins of the VV virion using 2-D gel electrophoresis followed by in-gel trypsin digests and peptide mass fingerprints for database searching [5]. Using a similar gel-based strategy, three major early proteins associated with the virosomes in VV-infected cells were identified by Murcia-Nicolas, *et al*. [6].

In this report we have utilized tandem mass spectrometry (MS) to analyze the protein composition of the vaccinia virion. A comprehensive proteome analysis of the protein composition of the VV virion represents an analytical challenge as there is no general analytical strategy available that is capable of identifying membrane and core proteins, low and high abundant proteins equally well. Therefore, we have used several analytical strategies to obtain a large number of high confidence protein identifications. Two different separation strategies [high performance liquid chromatography (HPLC) and sodium dodecyl sulfate-polyacrylamide gel electophoresis (SDS-PAGE)] were combined with tandem mass spectrometry. In addition, a "shotgun" approach with no further separation was evaluated. For the tandem mass spectrometry, three different MS instruments were utilized: 1.) a matrix assisted laser desorption ionization tandem mass spectrometer with time-of-flight/time-of-flight optics (MALDI-TOF/TOF), 2.) a quadrupole-time of flight mass spectrometer (LC-ESI-Q-TOF), and 3.) a quadrupole ion trap mass spectrometer (LC-ESI-QIT); the latter two instruments were equipped with online HPLC and electrospray ionization interfaces [7]. In the process of analyzing the vaccinia virion, we have identified sixty three VV proteins, two of which have not been reported previously.

## Results

### Viral fractionation

**Figure 1:**
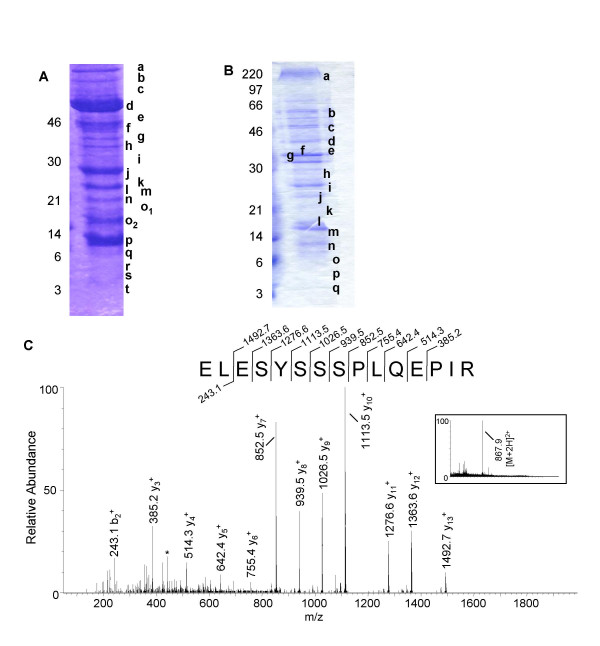
**Mass analysis of a distinct peptide from the L4R protein using Method 1 (SDS-PAGE + LC-ESI-Q-TOF MS) **Panel A shows the Coomassie blue stained SDS-PAGE gel of the core-enriched fraction and panel B is the membrane-enriched fraction. Gel slices that were analyzed by MS are denoted with letters. The full scan mass spectrum (inset of C) displays a doubly charged parent ion at m/z 867.9. The corresponding tandem mass spectrum (C) identifies a peptide of the L4R protein. Asterisks (*) denote the loss of ammonia (NH_3_) or water (H_2_O).

In order to simplify our analytical strategy, we partitioned the vaccinia virion into two enriched fractions: a supernatant or membrane fraction containing the soluble proteins and a fraction enriched with the cores and insoluble proteins. The fractionation was assisted by incubating purified virions in the presence of a reducing agent and non-ionic detergent. Beta-octylglucopyranoside (OG) was chosen as the detergent for dissolving the membrane because in low amounts it does not adversely affect MS analysis, whereas, conventional detergents such as SDS and Triton X100 can greatly interfere with HPLC and mass spectrometric analysis [8]. We tested the efficiency of OG in separating the virion components and found that the supernatant and pellet banding patterns on an SDS-PAGE gel differ (Figure 1A and 1B). Subsequent analysis of this separation with immunoblot analysis using antibodies to L1R (membrane protein) and 4b (A10L, core protein) showed that each fraction was enriched with these proteins (data not shown). Due to the comprehensive nature of this study, no attempts were made to completely separate the soluble membrane proteins from the core proteins.

### Identification of VV proteins

Table 1 summarizes the results of our proteomic study. Tandem mass spectrometry yields peptide sequences, allowing the search of non-redundant protein databases to obtain high confidence protein identifications. In total, over 2716 tandem mass spectra were analyzed to yield sequence information for 595 non-redundant peptides. Peptides scores of 40 or greater were considered positive matches. In rare cases, tandem mass spectra that yielded scores 20 and 40 were analyzed manually. In order for a protein to be a "positive" we used the following criteria: 1.) identify greater than 5% of the protein sequence; 2.) more than one peptide needed to be identified in a single method, or a single peptide needed to be identified at least with two different methods. Using these stringent conditions, sixty three different proteins were identified in the vaccinia virion. The total number of peptides found for each protein ranged from 1 to 62 (Table 1, column 5), and the total sequence coverage of the proteins ranged from 8.2% to 94.9% (Table 1, column 6). Of the sixty three proteins identified, 2 are predicted gene products that have not been shown to be expressed before: E6R and L3L (Table 1, italicized).

**Table 1:** Vaccinia virion proteins identified in this study. Membrane- and core-enriched fractions were both analyzed by five different methods: Method 1 (SDS-PAGE + LC-ESI-Q-TOF MS), Method 2 (SDS-PAGE + LC-ESI-QIT MS), Method 3 (HPLC + LC-ESI-QIT MS), Method 4 (LC-ESI-Q-TOF MS), and Method 5 (MALDI-TOF/TOF MS). Identified proteins are listed according to their corresponding ORF. The total number of non-redundant peptides and the percent of the protein identified are recorded.

ORF	Function/location	Ref.	Methods	# peptides	% Coverage
A3L	Major core protein	[17]	1,2,3,4,5	39	71.6
A4L	IMV/P4a associated protein	[18]	1,2,3,4,5	16	49.1
A5R	RNA pol. subunit	[19]	1,4,5	3	29.9
A7L	Early transcription factor	[20]	1,2,4,5	7	12.7
A10L	Major core protein	[21]	1,2,3,4,5	62	64.3
A12L	Viral structural protein	[22]	1,3,4	4	14.6
A13L	Membrane phosphoprotein	[23]	1,2,3,4,5	6	91.4
A14L	Membrane phosphoprotein	[23]	1,2,4,5	4	61.1
A15L	Core assoc. protein	[24]	1,2,3	5	60.2
A16L	Myristoylprotein; entry/fusion	[25, 26]	1,2,5	4	12.2
A17L	IMV membrane prtn	[27, 28]	1,2,4,5	4	32.0
A24R	RNA pol. subunit	[29]	1,2,4,5	26	30.9
A27L	IMV membrane prtn	[30]	1,2,3,4,5	17	70.0
A29L	RNA pol. subunit	[31]	2,5	2	8.2
A30L	Virion component	[32]	2,3,4,5	3	58.4
A33R	EEV glycoprotein	[33]	1,4,5	2	21.6
A34R	EEV glycoprotein	[34]	1,2,4	2	23.2
A42R	Profilin homolog	[35]	1,2,3,4,5	6	51.1
A46R	Interact with host IL-1	[36]	1	2	12.6
A56R	EEV glycoprtn, hemagglutinin	[37]	1,4,5	3	12.4
B5R	EEV glycoprotein	[38]	4	2	10.4
B22R	Serpin (C16L)	[39]	1,2,4,5	3	19.9
D1R	Capping enzyme subunit	[40]	1,2,4,5	15	22.7
D2L	virion component	[41]	1,2,4,5	9	63.0
D3R	virion component	[41]	1,2,4,5	8	50.6
D6R	Early transcription factor	[42]	2,5	7	11.9
D8L	IMV membrane protein	[43, 44]	1,2,3,4,5	26	89.1
D11L	DNA-dependent ATPase	[45]	1,2,5	9	17.3
D12L	Capping enzyme subunit	[46]	1,2,4,5	8	40.4
E1L	PolyA polymerase	[47, 48]	2,4,5	4	11.1
E3L	dsRNA dep. protein kinase	[49]	1,5	1	13.2
E4L	RNA polymerase	[50]	1,2,4,5	5	27.8
*E6R*	*unknown*		*1,2,4,5*	*20*	*43.1*
E8R	Virion component	[51, 52]	1,2,4,5	11	57.1
E10R	Oxidase	[53]	2,3,4,5	2	17.9
E11L	Viral core protein	[54]	1,2,4,5	2	26.4
F8L	Cytosolic protein	[55]	3,4,5	4	60.0
F9L	Mem. prtn.; similarity to L1R	[53]	1,2,3,5	4	22.6
F10L	Protein kinase	[56, 57]	1,2,5	4	15.7
F13L	EEV membrane protein	[58]	1,2,4,5	9	32.0
F17R	DNA binding phosphoprotein	[59]	1,2,3,4,5	9	55.4
G1L	metalloproteinase	[60]	1,2,4,5	10	19.1
G3L	Entry/fusion complex	[61]	2,3,4,5	6	41.4
G4L	glutaredoxin	[62]	2,3,4,5	11	77.4
G7L	Core cmpnnt, partners w/A30L	[63]	1,2,3,4,5	20	59.8
H1L	Protein phosphatase	[64]	1,2,3,4,5	10	67.3
H3L	Immunodominant protein	[65]	1,2,3,4,5	31	79.0
H4L	RNA pol. associated protein	[66]	1,2,4,5	5	10.6
H5R	Membrane phosphoprotein	[67]	1,2,3,4,5	8	49.3
I1L	encapsidated DNA-binding prtn	[68]	1,2,3,4,5	6	20.5
I3L	DNA binding phosphoprotein	[69, 70]	2,5	3	18.6
I5L	Virion component	[71]	1,4	5	94.9
I7L	Core protein proteinase	[72]	2	9	18.4
I8R	RNA/DNA-dependant NTPase	[73]	4,5	4	8.7
J1R	IMV membrane protein	[74]	1,2,4,5	5	30.1
J3R	Poly(A) polymerase, RNA methyltransferase	[48, 75]	1,2,4,5	14	47.4
J4R	RNA polymerase	[76]	1,2,4	6	38.4
J6R	RNA polymerase	[76]	1,2,4,5	34	33.9
K4L	Homolog to VP37, phoshoplipase D	[58, 77]	3,4	4	8.5
L1R	IMV membrane protein	[78]	2,3,4,5	8	40.8
*L3L*	*unknown*		*1,2,4,5*	*7*	*22.9*
L4R	Major core protein	[79]	1,2,3,4,5	25	77.7
O2L	Glutaredoxin	[80, 81]	1,2,3,4,5	7	70.4

### Method 1: SDS-PAGE + LC-ESI-Q-TOF MS

SDS-PAGE was employed to partition the core- and membrane-enriched fractions prior to MS analysis. The two protein fractions were resolved on a 12.5% SDS-PAGE gel and stained with Coomassie brilliant blue (Fig 1A and 1B). Each gel was sliced into several sections and each section was subjected to in-gel trypsin digestion as described in the Methods section. The tryptic digests were analyzed by LC-ESI-Q-TOF MS. As a typical example of the kind of data used for peptide identification using MASCOT software, the tandem mass spectrum of a peptide originating from the major core protein, L4R, is shown in Figure 1C. This spectrum was obtained from the tryptic digest of gel slice "h" (Fig. 1B). The full scan mass spectrum shows a peak at m/z 867.9 which represents the doubly charged ion of a peptide with a molecular mass of 1733.8 Da. Tandem MS of the doubly charged ion at m/z 867.9 yielded a fragment ion spectrum displaying eleven C-terminal (y-type) fragment ions and one N-terminal (b-type) fragment ion. Database searching of this tandem mass spectrum identified this peptide as ELESYSSSPLQEPIR, the partial sequence (amino acid [aa] 213–227) of the L4R protein. This tandem mass spectrum obtained the excellent score of 129. Using this method we obtained 708 spectra, observed 315 peptides and identified 52 proteins.

### Method 2: SDS-PAGE + LC-ESI-QIT MS

**Figure 2:**
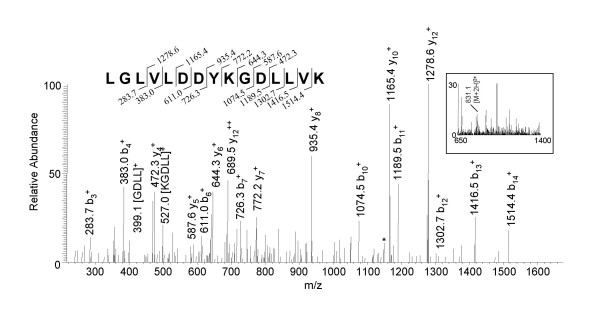
**Mass analysis of a distinct peptide from the E6R protein using Method 2 (SDS-PAGE + LC-ESI-QIT MS) **Gel slice "d" from the SDS-PAGE of the core-enriched fraction (Fig. 1A) was subjected to an in-gel trypsin digestion, and analyzed by LC-ESI-QIT MS. The tandem mass spectrum data, correlating to the full scan mass spectrum (inset, doubly charged parent ion at m/z 831.1), reveals a peptide of the E6R protein. Asterisks (*) denote the loss of ammonia (NH_3_) or water (H_2_O).

The tryptic digestions from the excised gel slices were additionally analyzed on an ion trap mass spectrometer (LC-ESI-QIT). Using this platform we identified 53 virion proteins from 1088 spectra corresponding to 417 total peptides. For example, during the mass spectrometric analysis of the tryptic digest of gel slice "d" (Fig. 1A) an ion peak at m/z 831.1 in the full scan mass spectrum was observed (Fig. 2C inset) which corresponds to a doubly charged ion of a peptide with molecular mass 1660.2 Da. The tandem MS of the double charged ion had a good score of 62 and revealed the sequence for a peptide of the E6R protein, LGLVLDDYKGDLLVK (aa 470–484). Seven C-terminal fragment ions, nine N-terminal fragment ions, and two internal fragments ions (m/z 399.1 [GDLL] and m/z 527.0 [KGDLL]) were observed for this particular peptide. E6R is a vaccinia protein that has not been previously reported.

### Method 3: HPLC + LC-ESI-QIT MS

**Figure 3:**
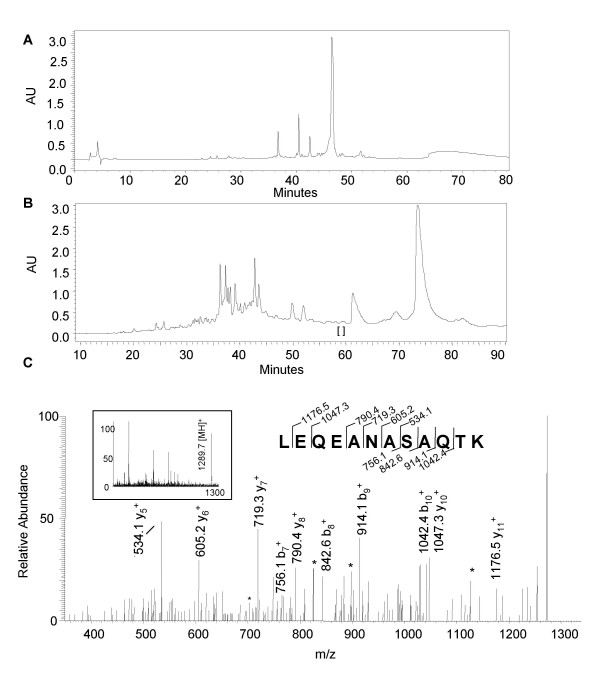
**Mass analysis of a distinct peptide from the L1R protein using Method 3 (HPLC + LC-ESI-QIT MS). **The core- (A) and membrane-enriched (B) fractions were resolved on a C_4 _HPLC column according to the Methods section. Tandem mass spectrometric analysis of fraction 59–60 (B, indicated by brackets) produced from a singly charged precursor ion (inset, m/z 1289.7), yielded fragment ions which corresponded to a peptide the L1R protein. Asterisks (*) denote the loss of ammonia (NH_3_) or water (H_2_O).

We also employed reverse phase HPLC to fractionate the proteins prior to MS analysis (Fig. 3A is the enriched core fraction and Fig. 3B is the enriched membrane fraction). HPLC separation was well suited for fractionating the soluble proteins, but proved to be more challenging for the insoluble core proteins. The cores did not completely dissolve even when treated with sodium deoxycholate. Approximately 200 μL of sample (as described in the Methods section) was loaded onto a 2 × 150 mm C_4 _reverse phase column, and fractions were collected manually every 2 minutes between 20 and 80 minutes. Each of these fractions was subjected to trypsin digestion prior to analysis by LC-ESI-QIT MS. Using this method we obtained 367 tandem mass spectra that correlated to 131 total peptides yielding 25 distinct vaccinia virion proteins. A representative example is shown in Figure 3C. The membrane sample at 59–60 minutes (Fig. 3B, brackets) underwent tandem mass spectrometric analysis to reveal a peptide of the well characterized L1R protein. The full scan spectrum for this fraction contained an ion at m/z 1289.7 (Fig. 3C, inset) which was used for tandem mass spectrometry. The fragment ions observed matched the theoretical fragmentation pattern for a peptide of the L1R protein (Fig. 3C) encompassing the sequence LEQEANASAQTK, aa 22–33. The ions at m/z 534.1, 605.2, 719.3, 790.4, 1047.3, and 1176.5, are the C-terminal fragment ions, while the ions at m/z 756.1, 842.6, 914.1, and 1042.4 are the N-terminal fragments. This spectrum received an acceptable score of 47.

### Method 4: LC-ESI-Q-TOF MS

**Figure 4:**
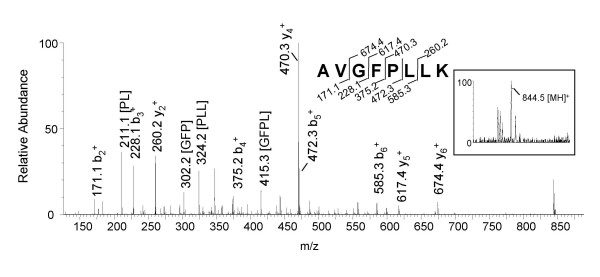
**Mass analysis of a distinct peptide from the L3L protein using Method 4 (LC-ESI-Q-TOF MS). **The core-enriched fraction of the virion was subjected to trypsin digestion, and analyzed by the LC-ESI-Q-TOF mass spectrometer. The full scan mass spectrum displays a peak at m/z 844.5 (inset), and corresponding tandem mass spectrum identifies a peptide of the L3L protein. Four internal fragments were also identified for the L3L peptide including: PL, GFP, PLL, and GFPL.

We wanted to analyze the samples without pre-fractionation to compare the data with thegel fractions (method 1 & 2) and HPLC fractions (method 3). Known as a "shotgun" approach, the membrane- and core-enriched fractions were directly digested with trypsin, and analyzed using LC-ESI-Q-TOF MS. This methodology resulted in 319 tandem mass spectra that matched 202 total peptides, and identified 53 virion proteins. One exciting example is the L3L protein (Fig. 4), a protein that has not been reported before. When the parent ion at m/z 844.5 (Fig. 4, inset) was fragmented, four C-terminal, five N-terminal, and four internal fragment ions (m/z 211.1, 302.2, 324.2, and 415.3) were observed. The respective tandem mass spectrum had a score of 59. This data was assigned to the sequence AVGFPLLK (aa 115–122) of the L3L protein.

### Method 5: MALDI-TOF/TOF MS

**Figure 5:**
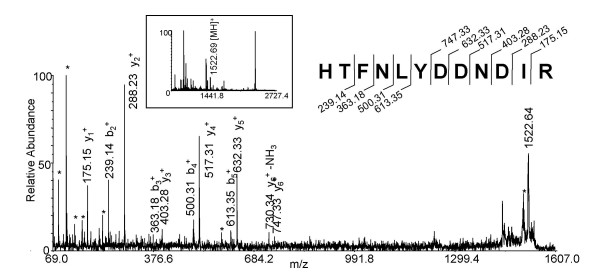
**Mass analysis of a distinct peptide from the G3L protein using Method 5 (MALDI-TOF/TOF). **The membrane-enriched fraction of the virion was subjected to trypsin digest, and analyzed by MALDI-TOF/TOF MS. The full scan mass spectrum yielded a singly charged ion at m/z 1522.69 (inset). Tandem mass spectrum of the parent ion corresponds to a peptide of G3L. Asterisks (*) denote the loss of ammonia (NH_3_) or water (H_2_O).

Direct trypsin digests of the membrane- and core-enriched fractions were also analyzed using MALDI-TOF/TOF MS to take advantage of complementary ionization techniques [7]. MALDI tandem mass spectrometry generated 234 spectra, correlating to 209 total peptides, and resulting in 55 unique virion protein identifications. Of particular interest is the ion at m/z at 1522.69 in the full scan mass spectrum (Fig. 5, inset). Tandem mass spectral analysis of this ion revealed the peptide HTFNLYDDNDIR, the partial sequence (aa 90–101) of the G3L protein. The tandem mass spectral analysis yielded six C-terminal, and 4 N-terminal fragment ions (Fig. 5), and obtained an average score of 41.

### New vaccinia virus proteins

This comprehensive study of the vaccinia virion revealed two newly observed proteins. Each of these proteins (E6R and L3L) has not been described previously. The peptides detected for each of these proteins are listed in Tables 2 and 3.

**Table 2:** Amino acid sequence of the VV protein E6R and identified peptides Peptides detected from the Method 1 (SDS-PAGE + LC-ESI-Q-TOF MS), Method 2 (SDS-PAGE + LC-ESI-QIT MS), Method 3 (HPLC + LC-ESI-QIT MS), Method 4 (LC-ESI-Q-TOF MS), and Method 5 (MALDI-TOF/TOF MS) are denoted with an asterisk (*). Peptides that are in bold print have been identified by at least one of the five methods.

Method	MDFIRRK**YLIYTVENNIDFLKDDTLSKVNNFTLNHVLALKYLVSNFPQHV**
1	***********************
2	******************** **********
3	
4	
5	
	**ITK**DVLANTNFFVFIHMVRCCKVYEAVLR**HAFDAPTLYVK**ALTKNYLSFS
1	*** ***********
2	*** ***********
3	
4	
5	
	NAIQSYKETVHKLTQDEK**FLEVAEYMDELGELIGVNYDLVLNPLFHGGEP**
1	
2	
3	
4	********************************
5	
	**IK**DMEIIFLKLFKKTDFKVVKKLSVIRLLIWAYLSKK**DTGIEFADNDRQD**
1	*************
2	
3	
4	**
5	**
	**IYTLFQQTGRIVHSNLTETFRDYIFPGDK**TSYWVWLNESIANDADIVLNR
1	**********
2	********
3	
4	
5	*********************
	**HAITMYDK**ILSYIYSEIKQGRVNKNMLK**LVYIFEPEKDIR**ELLLEIIYDI
1	************
2	********
3	
4	
5	
	PGDILSIIDAKNDDWKKYFISFYKANFINGNTFISDR**TFNEDLFRVVVQI**
1	*****
2	********
3	
4	********
5	*****
	**DPEYFDNERIMSLFSTSAADIK**RFDELDINNSYISNIIYEVNDITLDTMD
1	**********************
2	
3	
4	
5	*********
	DMKKCQIFNEDTSYYVKEYNTYLFLHESDPMVIENGILKKLSSIKSKSKR
1	
2	
3	
4	
5	
	**LNLFSK**NILKYYLDGQLAR**LGLVLDDYKGDLLVKMINHLKSVEDVSAFVR**
1	***************
2	****** *******************************
3	
4	
5	
	FSTDKNPSILPSLIKTILASYNISIIVLFQRFLR**DNLYHVEEFLDK**SIHL
1	************
2	
3	
4	
5	
	TKTDKK**YILQLIR**HGRS
1	
2	
3	
4	
5	*******

**Table 3:** Amino acid sequence of the VV protein L3L and identified peptides Peptides detected from the Method 1 (SDS-PAGE + LC-ESI-Q-TOF MS), Method 2 (SDS-PAGE + LC-ESI-QIT MS), Method 3 (HPLC + LC-ESI-QIT MS), Method 4 (LC-ESI-Q-TOF MS), and Method 5 (MALDI-TOF/TOF MS) are denoted with an asterisk (*). Peptides that are in bold print have been identified by at least one of the five methods.

Method	MNTR**TDVTNDNIDKNPTK**RGDRNIPGRNERFNDQNRFNNDRPRPKPRLQP
1	**************
2	**************
3	
4	**************
5	
	NQPPKQDNKCREENGDFINIRLCAYEKEYCNDGYLSPAYYMLKQVDDEEM
1	
2	
3	
4	
5	
	SCWSELSSLVRSRK**AVGFPLLK**AAKRISHGSMLYFEQLKNSKVVKLTPQV
1	
2	
3	
4	********
5	
	KCLNDTVIFQTVVILYSMYKRGIYSNEFCFDLVSIPRTNIVFSVNQLMFN
1	
2	
3	
4	
5	
	ICTDILVVLSICGNRLYRTNLPQSCYLNFIHGHETIARR**GYEHSNYFFEW**
1	
2	
3	
4	
5	***********
	**LIKNHISLLTKQTMDILK**VKKKYATGAPVNRLLEPGTLVYVPKEDYYFIG
1	
2	***************
3	
4	*******
5	***
	ISLTDVSISDNVR**VLFSTDGIVLEIEDFNIKHLFMAGEMFVR**SQSSTIIV
1	
2	
3	
4	******************
5	*****************************

The E6R ORF is situated between the E5R and E7R genes and produces a 567 amino acid protein. The predicted molecular mass and pI of E6R is 66,670 Da and 6.16, respectively. E6R was identified in fraction "d" of figure 1A, which corresponds to its predicted molecular weight. Blast searches revealed high homology to orthopoxvirus proteins [9]. Hydrophobicity plots revealed no specific region of interest [10]. We observed 19 peptides from the E6R protein with a confidence scoring range of 17–85. The identified peptides covered 43.1% of the protein (Table 2).

We observed 7 peptides for L3L covering 22.9% of the sequence (Table 3). The L3L protein has a predicted molecular mass of 40.6 kDa (350 amino acids), and a predicted pI of 8.91. Its ORF is situated between the L2R and L4R genes. This protein was identified in fraction "e" and "f" of Figure 1A. Only poxvirus proteins had homology to the L3L sequence resulting from Blast searches [9], and hydrophobicity plots revealed no specific region of interest [10].

Both proteins were found in samples from the core-enriched fractions of Method 1, 2, 4, and 5. No peptides from either protein were found in the membrane-enriched fractions.

## Discussion

The goal of this study was to obtain a comprehensive proteomic analysis of the Copenhagen strain of the vaccinia virus virion. This strain of VV was chosen because it is an important model strain for variola, and it has been completely sequenced.

**Figure 6:**
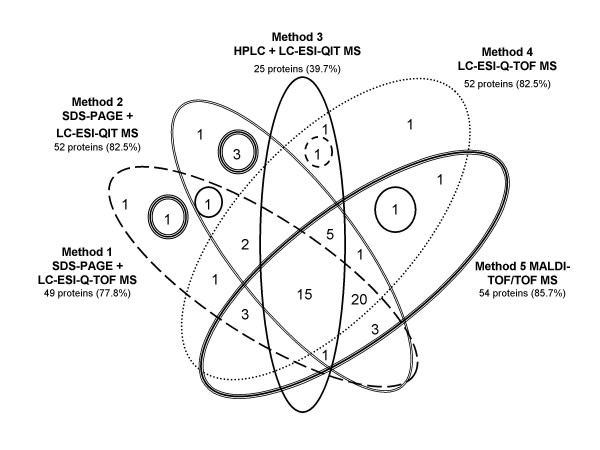
**Diagram of proteins identified using multiple methods. **All overlaps are shown for all five methods: Method 1 - SDS-PAGE + LC-ESI-Q-TOF MS (dashed line); Method 2 - SDS-PAGE + LC-ESI-QIT MS (double line); Method 3 -HPLC + LC-ESI-QIT MS (solid line); Method 4 - LC-ESI-Q-TOF MS (dotted line); and Method 5 - MALDI-TOF/TOF (triple line). Numbers represent the number of shared proteins in overlapping areas. The total number of proteins identified for the method and the percent of total proteins identified is listed.

One concern we had was that the predominant proteins would eclipse the smaller or less abundant proteins when analyzed by MS. In order to overcome this problem we fractionated the virion into soluble (membrane) and insoluble (core) fractions via treatment with detergent and centrifugation. Further fractionation was achieved using two procedures: SDS-PAGE and HPLC. The resolution of viral proteins by SDS-PAGE followed by in-gel trypsin digestion of gel slices and tandem mass analysis (LC-ESI-QIT MS) for protein identification had been used successfully before on other VV proteins [11]. A second MS analysis was done in parallel with these samples using LC-ESI-Q-TOF MS. Although both instruments use the same ionization techniques, the mass analyzers are different. Both mass spectrometers identified 49–52 proteins using this procedure, however, the proteins identified differed (Fig. 6). Complementary to SDS-PAGE for protein fractionation, reverse phase HPLC was used (Method 3). Due to the encountered difficulties with the insolubility of the viral cores, only the major core proteins were identified (A3L and A10L) from the core-enriched fraction, resulting in a low total number of proteins identified with this procedure (25 versus 49–54 for the other methods, Fig. 6). Support for this notion is obtained by the study reported by Zachertowska, *et al*. in which the pooling of fractions from 5 HPLC runs resulted in the identification of only 6 proteins of the myxoma virion [12]. Recognizing this limitation we utilized multiple methods to obtain a more comprehensive catalog of the virion constituents. In order to complete this study, we felt it important to analyze the membrane- and core-enriched samples without separation prior to trypsin digestion. We used two different mass spectrometers to analyze the in-solution digests: MALDI-TOF/TOF MS and LC-ESI-Q-TOF MS. This "shotgun" strategy resulted in a lower number of total spectra and identified a lower number of peptides, but yielded a comparable number of protein identifications (54 and 52, respectively, Fig. 6).

A summary of the number of proteins found versus the method used to detect them is shown in Figure 6. There is a high degree of overlap between the methods; noteworthy is that 15 proteins were identified by all 5 methods. Another 20 proteins were identified using methods 1, 2, 4, and 5; this is most likely due to the lack of data for the core-enriched fraction using the HPLC pre-separation procedure (method 3). The majority of the VV proteins identified in this study were observed in 3 or more methods (85.7%), underscoring the complementarity of the different approaches used.

The current functional annotation of the VV genome is described in the following articles: a minireview by Paoletti, *et al *[4], describing an update on the vaccinia genome, and the Poxviridae chapter in Fields Virology written by Bernard Moss [3]. Both of these articles describe the organization of the entire genome of the vaccinia virion, and the known functionality of the various vaccinia proteins. Moss describes there being 47 known ORFs that express proteins of the vaccinia virion including membrane proteins as well as core constituents. It is interesting to note that we found 41 of the known virion components. Of the 25 non-enzymatic components only one was not identified – the D13L protein which has been linked to rifampicin resistance. Of the 22 enzymatic virion components 17 were identified in this study. Two of the missed proteins include D7R and G5.5R which are the two smallest subunits of the RNA polymerase. Although these two components were not identified, the other six RNA polymerase subunits were identified (A5R, A24R, A29L, E4L, J4R and J6R). The remaining three known virion enzymes that were not identified in this study include: A18R (DNA-dependent ATPase), B1R (Protein Kinase 1) and H6R (DNA Topoisomerase 1). Several factors might contribute to the lack of data for these proteins including: the size of the protein, the hydrophobicity of a protein, and the absolute amount of a protein in the virion. In general, very hydrophobic proteins and low abundance proteins are commonly underrepresented in proteomic-type studies. Also, very small proteins are frequently missed. In an effort to overcome at least in part these inherent limitations of comprehensive proteomic studies, we combined different protein fractionation methods with "shotgun" approaches. In addition, to ensure that the highest level of confidence for peptide identification and protein coverage for the current study, the "shotgun" digests were analyzed by two different ionization techniques, ESI and MALDI, taking advantage of the complementarity of these ionization techniques [7].

**Figure 7:**
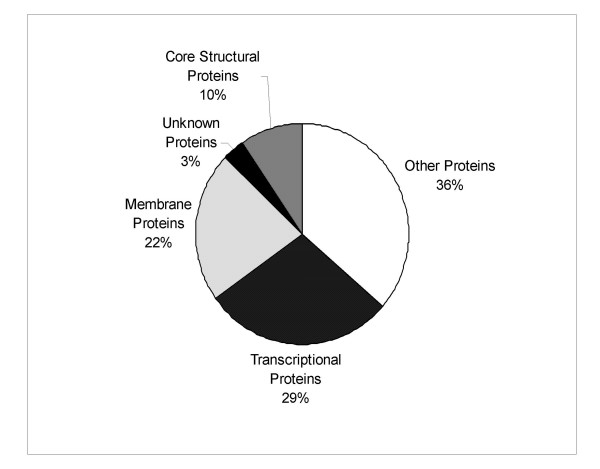
**Summary of the functions of the 63 identified VV proteins. **This graph summarizes the 63 VV proteins identified in this study according to their function. These include: core structural proteins (A3L, A4L, A10L, A12L, A14L, and L4R), membrane proteins (A13L, A17L, A27L, A33R, A34R, A56R, B5R, D8L, F9L, F13L, H3L, H5R, J1R, and L1R), transcriptional proteins (A5R, A7L, A24R, A29L, D1R, D6R, D11L, D12L, E1L, E3L, E4L, H4L, I1L, I8R, J3R, J4R, J6R, and K4L), proteins with other functions (A15L, A16L, A30L, A42R, A46R, B22R, D2L, D3R, E8R, E10R, E11L, F8L, F10L, F17R, G1L, G3L, G4L, G7L, H1L, I3L, I5L, I7L, and O2L), and the unknown proteins (E6R and L3L).

Some recently reported proteins that have been shown to be associated with the core include: A15L, A30L, E11L, G1L, G7L, H1L and J1R – all of which were identified in our analysis. We also found membrane proteins (F9L, F10L, and E8R) and cytosolic proteins (A16L, E10R, F8L, G4L, and I3L). The remaining proteins identified by this study included A42R, A46R, B22R, E3L, and K4L. The types of VV proteins identified in this study are summarized in Figure 7.

## Conclusion

This study represents the first steps toward a widespread identification of the viral protein constituents that make up the structurally complex VV virion. Although not quantitative, this approach has confirmed the presence of most known virion proteins and identified new viral proteins for the first time. Defining the VV proteome is a significant scientific challenge but successful completion of this goal will be useful for understanding the biology of orthopoxviruses.

## Methods

### Materials

Media and supplements were purchased from Invitrogen (Carlsbad, CA). Dithiothreitol (DTT), Tris, N-ethylmorpholine, trifluoroacetic acid (TFA), formic acid, α-cyano-4-hydroxycinnamic acid (HCCA), guanidine hydrochloride and β-Octylglucopyranoside (OG) were purchased from Sigma (St. Lois, MO). HPLC grade acetonitrile (AcN) was purchased from Fisher Scientific (Pittsburg, PA) and the lyophilized sequencing-grade trypsin from Promega (Madison, WI). General use water was generated by a Milli-Q water purification system (Millipore, Bellerica, MA)

### Virion purification and fractionation

Vaccinia virus (Copenhagen strain) was propagated as described by Hruby *et al*[13], and modified in the following way: ten 150 mm dishes of 80% confluent BSC_40 _cells were infected at a multiplicity of infection of 0.1 plaque forming units (pfu)/cell. The infected cells were harvested and subjected to homogenization. Purified virus was obtained through a side-band-pull from a buoyant sucrose density gradient. This purified virus was pelleted by centrifugation. To obtain the membrane- and core-enriched fractions, a protocol from Spencer, *et al*[14] was modified. The equivalent of three tubes (or thirty 150 mm dishes) was resuspended in 300 μL of buffer (0.8% OG, 50 mM DTT, 50 mM Tris, pH = 8.4). The three tubes containing the pelleted virus were rinsed with another 300 μL of buffer. The total 600 μL was placed in an incubator shaker (New Brunswick, Innova 4300, Edison, NJ) for 1.25 hours, shaking at 200 rpm at 37°C. The sample was centrifuged at 15,000 × g at 4°C for 15 min. The supernatant containing the enriched membrane fraction was extracted leaving the pellet (enriched core fraction) intact.

### HPLC (membrane-enriched fraction)

The sample was transferred into a dialysis cassette (Pierce, Slide-A-Lyzer, 3,500 Da molecular weight cut off, MWCO) and dialyzed against 800 mL of 20 mM Tris (pH=8.0) for 2 hours at room temperature, and changed three times. The final dialysis was performed for 16 hours at 4°C. Dialysis was performed to remove as much of the OG as possible. The sample was removed from the cassette, reduced to 500 μL using a speed-vac, and filtered through a 0.2 μ syringe filter. The sample was injected onto an HPLC (Waters Breeze system, Milford, MA) using a 500 μL sample loop. A 5 μm C_4 _300 Å 2 × 150 mm column (Phenomenex Jupiter, Torrance, CA) was used with a flow rate of 0.2 mL/min. A wavelength absorbance detector (Waters 2487 Dual Absorbance, Milford, MA) was used to detect wavelengths at 214 and 254 nm. Solvent A was composed of 5% AcN, 0.05% TFA in H_2_O and solvent B was composed of 95% AcN, 0.05% TFA in H_2_O. For the membrane-enriched sample the gradient system consisted of 10 min isocratic gradient with 98% of solvent A, 55 min gradient to 2% A, and 10 min isocratic gradient with 2%. Fractions were collected in 5 minute intervals for the first 20 min and then in 2 min intervals for 60 min. The final 7 min was collected in one final fraction. A speed-vac was used to decrease the volume of the fractions to about 5–10 μL. The pH was adjusted with 50 mM Tris (pH = 9.6) or N-ethylmorpholine to between 7.0 and 8.5. For the trypsin digest 1.5 μL of trypsin (1 μg/μL, Promega, Madison, WI) was added to each fraction. The fractions were incubated overnight at 37°C.

### HPLC (core-enriched fraction)

The core-enriched fraction was dissolved prior to injection on the HPLC [12]. Briefly, this was carried out by resuspending the insoluble pellet from the virion fractionation in 0.4% sodium deoxycholate and 10 mM Tris, pH = 9.0, and incubating at 56°C for 10 min. The sample was filtered and injected onto the HPLC system as described above. For the core-enriched sample the gradient system consisted of a 10 min isocratic gradient with 98% of solvent A, 40 min gradient to 25%A, 30 min gradient to 2% A, and a 10 min isocratic gradient with 2% A. Fractions were collected as outlined above.

### SDS-PAGE

Virion fractions were mixed with equal amounts of SDS-PAGE loading buffer and resolved on SDS-PAGE gels (12.5%) [15]. The protein bands were visualized using a filtered Coomassie stain (40% methanol, 10% acetic acid and 0.2% Coomassie brilliant blue R-250). Protein bands were excised and the preparation for in-gel digestion [16] was as follows: sample bands from coomassie stained were manually excised, and placed in 0.5 mL Eppendorf tubes. The gel pieces were de-stained with acetic acid:ethanol:H_2_O in a 1:3:6 (v/v/v) ratio for 12–16 hours under gentle agitation. The gel slices were dehydrated with AcN (vortexed for 10 min), and re-hydrated with 50 mM ammonium bicarbonate (vortexed for 10 min). This procedure was repeated twice, and a final dehydration was performed with AcN. After vortexing any remaining liquid was removed by pipette and the gel slices were dried with a speed vac for 5 minutes.

### In-gel trypsin digestion

To each tube containing gel slices 10–40 μL of 20 μg/μL Promega trypsin in 25–50 mM Tris-HCl, pH = 8.0 was added. The tubes were incubated on ice for 35–40 min. After the enzyme solution was fully absorbed, the excess solution was removed and replaced with 10 mM Tris-HCl, pH = 8.0, enough to fully cover each gel slice. Each sample was incubated at 37°C for 12–16 hours. Any non-absorbed solution was placed in a new tube. The peptides were extracted from the gel by vortexing with 30–70 μL of 50% AcN/5% formic acid. The extraction fluid was added to the previously removed non-absorbed solution and concentrated to 10–15 μL.

### In-solution trypsin digestion

Samples from the HPLC or from the membrane-enriched fraction were subjected to in-pot trypsin digest by adding a 1:7 ratio of enzyme (1 μg/μL):protein and incubating at 37°C for 12–16 hours. Because the core-enriched fraction contained an intact protein ''shell'' the following was conducted in order to solubilize the proteins for trypsin digestion. The pellet from the virion fractionation was resuspended in 200 μL of 50 mM Tris (pH = 8.4), and 6 M guanidine HCl. This solution was brought to 90°C for 5 minutes, and after the protein was dissolved, the mixture was diluted with 2.8 mL of H_2_0. Dialysis was performed with a Slide-A-Lyzer (3,500 MWCO) membrane against 800 mL of 20 mM Tris-HCl (pH = 8.0). The buffer was changed 3 times in 24 hours. As the guanidine HCl dialyzed out of the protein solution the proteins precipitated resulting a fluffy white precipitate. However, when this solution was treated with trypsin (as outlined above) the precipitate dissolved indicating that the trypsin cleaved the proteins into various peptides.

### LC-ESI-QIT MS

An electrospray ionization quadrupole ion trap mass spectrometer (Finnigan LCQ, San Jose, CA) equipped with a Waters (Milford, MA) 515 HPLC system and LC Packings Accurate Flow Splitter was used. Ten microliters of the tryptic digest was loaded on a C_18 _trap and a C_18 _column (0.17 × 10 mm, Jupiter 5 μ, 300 Å, packed in-house). HPLC was performed with a gradient from 90% solvent A (0.1% formic acid, 0.005% TFA, in 5% AcN) to 90% solvent B (0.1% formic acid, 0.005% TFA in 95% AcN) over 60 minutes. The full mass spectra (m/z 400 to 2000) and tandem MS (m/z 200 to 2000) spectra were acquired alternately with a dynamic exclusion of 1 min and the peptide was excluded for 1.5 min.

### LC-ESI-Q-TOF MS

Five microliters of the tryptic digest sample was mixed with 5 μL of solvent A (0.1% formic acid, 0.005% TFA, and 3% AcN in H_2_O). Five microliters of this solution was loaded for mass analysis. The HPLC was performed on a Waters CapLC system with a flow rate of 300 nL/min in conjunction with a Symmetry 300, C_18, _5 μm trap from Waters (Milford, MA) and a 15 cm long, 75 μm inner diameter PicoFrit column from New Objective (Woburn, MA) packed in-house with Jupiter C_18 _particles from Phenomenex (Torrance, CA). The gradient program began with 3% B (0.1% formic acid, 0.005% TFA in 90% AcN) for 5 min to wash the sample, followed by a gradient up to 30 % B over 40 min, to 50 % B at 60 min, to 70% B at 65 min, and held at 90% B from 70 to 78 min. The LC-ESI-Q-TOF mass spectrometer (Global Ultima; Micromass, Ltd., Manchester, UK) was used with a spray voltage of 3.5 kV. The MS/MS data was recorded using a 0.5 sec MS survey scan and 2.5 sec MS/MS scans on the three most abundant ions found in the survey scan. The CID energy was between 25 and 65 eV depending on the mass and charge state of the precursor ions.

### MALDI-TOF/TOF MS

The in-pot tryptic digest sample was loaded on a Symmetry 300 C_18 _trap and a 150 mm by 0.32 mm Symmetry column both packed with 5 μm C_18 _particles from Waters for off-line HPLC separation before the mass analysis. The same gradient was used as in the LC-ESI-Q-TOF MS with solvent A (0.1% TFA and 1% AcN in H_2_O) and solvent B (0.1% TFA and 1% H_2_O in AcN) with a flow rate of 3 μL/min from a Waters CapLC system. The elutant from the column was automatically mixed with 1 μL/min of 0.6 mg/mL HCCA and 0.08 mg/mL ammonium phosphate in 50:50 AcN:H_2_O containing 0.1% TFA. The sample was passed through a 75 μm capillary at a combined rate of 4 μL/min into a Waters MALDIprep sprayer (Life Sciences R & D Laboratory, Waters Corporation). A spotting time of 45 second per spot, a nitrogen flow rate of 7 psi, and a temperature gradient of -2°C/7 min (65°C to 45°C over 90 min) was used. For MALDI tandem mass spectrometry an Applied Biosystems 4700 proteomics Analyzer (Applied Biosystem, Inc., Framingham, MA) was used. MS data were acquired using the reflector mode. Ten tandem mass spectra were recorded from each spot. Ions with a signal to noise (S/N) ration greater than 30 were chosen for tandem mass spectrometry. The tandem mass spectra were recorded by accelerating the precursor ions to 8 keV, selecting with the timed gate set to 3 Da, and performing the collision induced dissociation (CID) at 1 keV. The gas pressure (air) in the CID cell was at 6 × 10^-7 ^Torr and the fragment ions were accelerated to 14 keV before entering the reflector.

### Data Analysis

Mascot (Matrix Science, London, UK) software was used for the protein identification. The uninterpreted tandem mass spectral data were searched against a VV protein database created in-house based on the complete DNA sequence of vaccinia virus (PUBMED 2219722). Specifically, 273 protein sequences in FASTA format were organized and loaded onto the in-house Mascot primary sequence database. All MS data was also searched against the MSDB database, a composite, non-identical protein sequence database built from a number of primary source databases (Matrix Science). The LC-ESI-QIT MS data was converted into Sequest DTA files and searched with the Mascot program.

## Competing interests

The author(s) declare that there are no competing interests.

## Authors' contributions

JDY conceived of the study, participated in its design and coordination, assisted with sample preparation, and drafted the manuscript. CRG assisted with the propagation of virus, prepared the samples for analysis, and helped analyze the mass spectrometry data. TSC prepared samples for analysis, analyzed the mass spectrometry data and helped to draft the manuscript. SV assisted with the analysis of the mass spectrometry data. DEH and CSM coordinated the research efforts and edited the manuscript. All authors read and approved the final manuscript.
